# Translation and validation of the Second Victim Experience and Support Tool-Revised into Brazilian Portuguese

**DOI:** 10.1590/1806-9282.20241154

**Published:** 2024-12-02

**Authors:** Ana Paula Almeida Lima, Marcos Antônio Almeida Matos, Carolina Villa Nova Aguiar

**Affiliations:** 1 Escola Bahiana de Medicina e Saúde Pública – Salvador (BA), Brazil.

**Keywords:** Medical error, Validation study, Occupational health, Mental health

## Abstract

**INTRODUCTION:**

The term second victim arises when a healthcare professional experiences an adverse event that has an emotional impact and/or physical suffering for the healthcare professional. The experiences of the second victim can be measured using the Second Victim Experience and Support Tool, originally developed in English, translated and validated internationally in several languages. A revised version of the tool (Second Victim Experience and Support Tool-Revised) has been published since 2020.

**OBJECTIVE:**

The objective of this study was to translate and gather evidence of the validity of the Second Victim Experience and Support Tool-Revised Version into Brazilian Portuguese.

**METHODS:**

This is a methodological study that involved the necessary procedures for the translation and cross-cultural adaptation of the instrument, carefully following the stages of translation, back-translation, pre-testing, evaluation by a panel of experts, and a test on a large population for validity and reliability.

**RESULTS:**

A total of 146 health professionals took part in the study, with a greater predominance of doctors (38.4%) and physiotherapists (30.8%). Seven items were excluded, all with factor loadings of less than 0.50. The "institutional support" dimension had to be excluded due to its low factor loadings. The fit indices of the re-specified model were χ^2^/df=1.60, CFI=0.90, TLI=0.88, and RMSEA=0.07 (CI 0.06–0.08).

**CONCLUSION:**

The adapted version of the instrument showed satisfactory psychometric properties when composed of 28 items distributed in eight dimensions, making it a valid and reliable instrument for assessing second victim experiences in different professions, suitable for dissemination in Brazil.

## INTRODUCTION

The World Health Organization (WHO) classifies adverse events as incidents that result in unintentional harm arising from care and not related to the natural course of the patient's underlying disease. In addition, according to the WHO, of the 421 million hospitalizations that take place every year in the world, almost 10%—42.7 million—are recorded as adverse events, ranging from mistaken administration of medication to damage that leads to the death of patients^1^.

In the Global Action Plan for Patient Safety 2021–2030, published by the WHO, it is estimated that around 1 in 10 patients in high-income countries experience adverse events when receiving hospital care. For low- and middle-income countries, this proportion rises to 1 in 4 patients, generating approximately 134 million adverse events per year, contributing significantly to around 2.6 million deaths^2^.

Adverse events also cause suffering for healthcare professionals, often exposing them to the position of being solely responsible for their occurrence, and sometimes few or no measures are implemented to address these issues in healthcare organizations, exposing professionals to a punitive culture in the institutional environment^3^. The term "second victim" first appeared in an editorial in the British Medical Journal, which discussed the impact of medical errors on the professionals involved^4^. The concept was introduced by Albert Wu, describing that health professionals also face trauma as a result of their exposure to unavoidable adverse events, unable to cope with the situation^5^. Despite the prevalence and consequences of this phenomenon, it is still not widely understood among health professionals^6^.

The Joint Commission encourages healthcare institutions to support second victims as soon as an adverse event occurs. It argues that by offering a support option to traumatized healthcare professionals, organizations can help prevent the domino effect that adverse events can have on the performance of these professionals, which in turn can have a direct impact on the care that this professional provides to other patients^7^. It is important to emphasize that implementing support measures for health professionals directly involved in errors does not imply exemption from their responsibility since this does not reduce the damage caused to the first victim (the patients). However, it is a welcome to the professional, aimed at preventing the error from being repeated and allowing it to continue in the work environment^8^.

The Second Victim Experience and Support Tool (SVEST)^9^, originally published in English, was developed to help assess and intervene with second victims. This same tool was later validated and translated into other languages^10–16^. Since 2021, a revised version of the instrument (SVEST-R) has been available in English^17^ and translated into German^6^ and Malaysia^18^. In Brazil, we did not identify in the literature the translation and validation of the SVEST-R. This study aims to translate and gather evidence of the validity of the SVEST-R into Brazilian Portuguese.

## METHODS

This is a methodological study to translate and validate the SVEST-R into Brazilian Portuguese.

Initially, authorization was requested from the scale's developer, Dr. James M. Hoffman, with an affirmative response for translation and cross-cultural adaptation. The English version was then translated into Brazilian Portuguese. At this stage, we had two independent, bilingual translators, generating two versions of the instrument. Once the two translations were available, the research team carried out a comparative analysis, generating Synthesis I of the instrument. Synthesis I was then back-translated by two new independent translators who were native English speakers and fluent in Portuguese. These researchers/translators were not familiar with the terms used in the scale and did not have access to the original English version of the instrument. The two versions of the back-translation were analyzed again by the researchers and compared in terms of the format, wording, and grammatical structure of the sentences. Synthesis II was produced from this comparison.

Synthesis II was then pilot-tested with a sample of 10 health professionals. Each professional evaluated the items on the scale in terms of clarity, comprehension, and appropriateness. To do this, a Likert-type scale was used, with scores ranging from 1 to 5, where the closer to 5 the better the item was assessed. Next, the content validity coefficient (CVC) was calculated, and the items that obtained a CVC below 0.8 were re-analyzed and the necessary modifications were made, giving rise to the version of the instrument that supported the psychometric testing stage on a sample of the target population.

Health professionals were recruited nationwide for this stage. The inclusion criterion was being a healthcare professional involved in direct patient care, and the exclusion criterion was not having been involved in a previous adverse event. Data were collected using an online survey with snowball sampling. The analyses were carried out using confirmatory factor analysis (CFA). Bootstrapping procedures were carried out (200 re-samples) to achieve greater reliability of the results and reduce deviations from the normal distribution of the sample. The adequacy of the model tested was observed using the fit indices: estimate of χ^2^ divided by the degrees of freedom (χ^2^/df) (values less than 3 are considered adequate), root mean square error of approximation (RMSEA) (values less than 0.08 were considered satisfactory), comparative fit index (CFI), and Tucker-Lewis index (TLI) (values greater than 0.90 were considered satisfactory)^19^. To make adjustments to the model, the factor loadings (items with factor loadings below 0.50 were excluded) and the suggested modification indices were observed. To analyze the reliability, composite reliability indices were calculated, with values above 0.8 being considered satisfactory^20^.

This study was submitted to the Research Ethics Committee of the Bahiana School of Medicine and Public Health and was approved under protocol number 5,548,533.

## RESULTS

A total of 146 health professionals took part in the study, with doctors (38.4%) and physiotherapists (30.8%) predominating. The median age of the professionals was 38.0 (IQR: 34.0–43.2) years, and the median length of experience was 12.0 (8.0–18.0) years.

**Figure 1:**
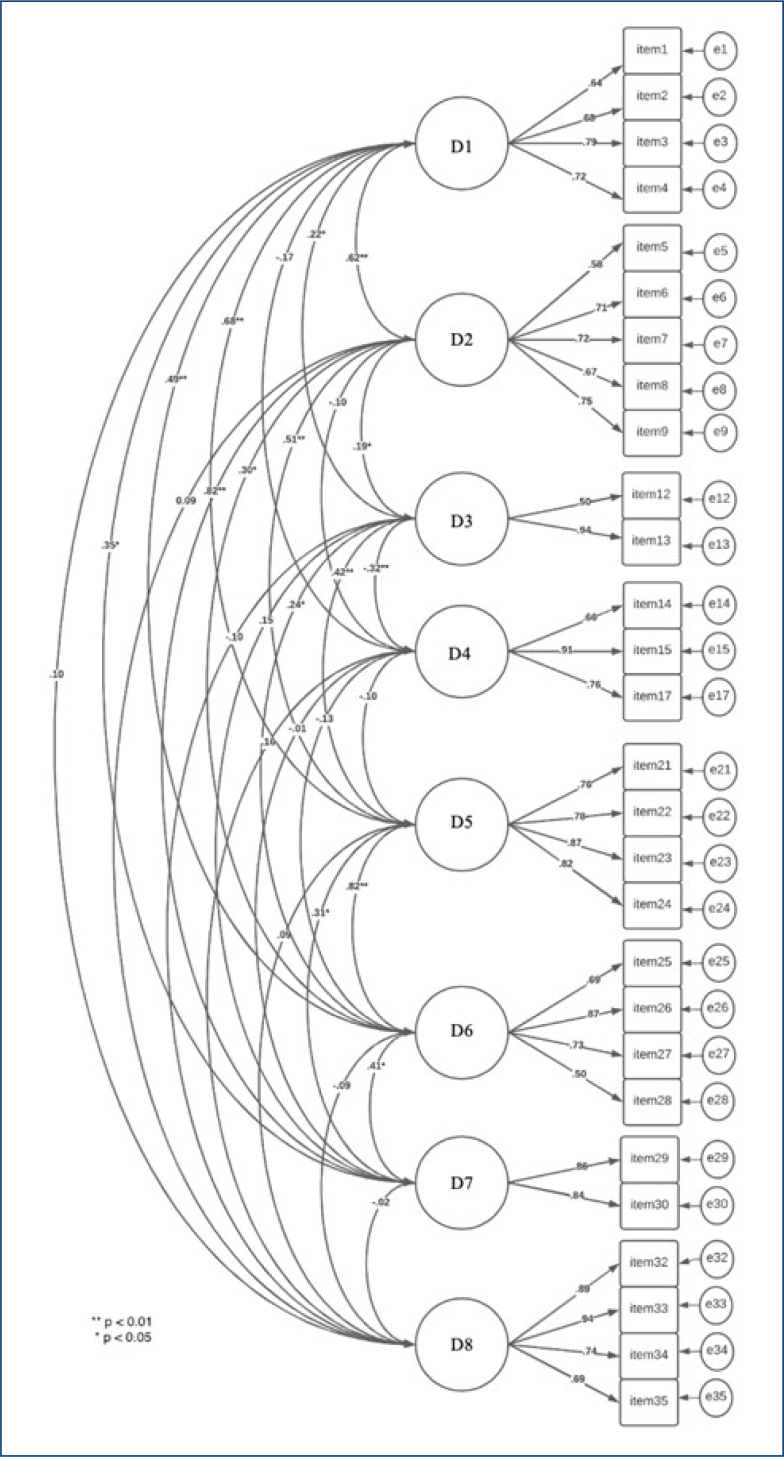
Re-specified Second Victim Experience and Support Tool-Revised model. Notes: D1: psychological problems; D2: physical problems; D3: support from colleagues; D4: support from supervisors; D5: professional self-efficacy; D6: intention to change; D7: absenteeism; D8: resilience.

The hypothetical measurement model for the CFA was specified according to Winning's proposal^21^, that is, the 35 items were distributed across the nine dimensions. The model's fit indices were: χ^2^/df=2.03, CFI=0.76, TLI=0.77, and RMSEA=0.08 (CI 0.08–0.09). Seven items were excluded, all with factor loadings of less than 0.50. It should be noted that the "institutional support" factor had to be excluded as a result of the exclusion of items with low factor loadings. After analyzing the modification indices, it was decided not to insert new parameters into the model. The fit indices of the re-specified model were χ^2^/df=1.60, CFI=0.90, TLI=0.88, and RMSEA=0.07 (CI 0.06–0.08) (Figure 1).

The composite reliability indices for the eight dimensions ranged from 0.70 to 0.89, as shown in Table 1.

**Table 1:** Composite reliability indices of the eight dimensions of the Second Victim Experience and Support Tool-Revised, n=146, Salvador/BA, Brazil.

Dimension	Items	Composite reliability
Psychological problems	4	0.80
Physical problems	5	0.81
Support from colleagues	4	0.70
Support from supervisors	4	0.79
Professional self-efficacy	4	0.89
Intention to change	4	0.80
Absenteeism	3	0.84
Resilience	4	0.89

## DISCUSSION

This is the first study to translate and cross-culturally adapt the SVEST-R into Brazilian Portuguese. We identified the existence of a previous translation of the SVEST in Brazil^22^. However, this translation did not include the additional dimensions present in the SVEST-R. In addition, our research did not identify any studies testing the psychometric evidence of the instrument in Brazil. For these reasons, this study opted to carry out the complete process of translation and cross-cultural adaptation, carefully following all the recommended steps^23^.

From a psychometric point of view, the decision was made to use CFA since it is an instrument that follows an established theoretical model and has been previously tested in other countries and contexts^6,18^. Also for these reasons, the choice was made to keep only those items with high factor loadings (above 0.50), which led to the exclusion of seven items and, consequently, the need to exclude the "institutional support" dimension. It is possible to raise a number of hypotheses for this result in Brazil, the most accepted by the authors of this research being that institutional support for second victims in Brazil is still a subject under development, little explored, where there is a significant gap between the content produced internationally and that produced in our country^8^.

When evaluating the fit indices obtained in other studies, it can be seen that there was also a need to make changes to obtain a psychometrically satisfactory model. The validation study in Malaysia^18^, for example, presented a final model made up of seven dimensions: the psychological problems and physical problems dimensions were combined into one (called "stress"), as were the intention to change and absenteeism dimensions (which were renamed "negative consequences"). In addition, the model excluded three items, which were also excluded in the final version of this study. In the German validation^6^, the final instrument was made up of five factors (stress, support, change, resilience, and request for support).

To assess the internal consistency of the dimensions, composite reliability indices were calculated. Although Cronbach's alpha index is still more widely known and used as a consistency indicator, some restrictions to its use have been pointed out in the literature (especially the fact that it is strongly affected by the length of the test)^24^. For this reason, and especially considering the existence of dimensions with only two items in the instrument, we opted to adopt the composite reliability index, which has been presented as a more robust indicator of accuracy^25^. All the dimensions obtained indices were considered satisfactory (above 0.70), with the majority being excellent (above 0.80).

## CONCLUSION

The adapted version of the instrument showed satisfactory psychometric properties when composed of 28 items distributed over eight dimensions, making it a valid and reliable instrument for assessing second victim experiences in different professions, suitable for dissemination in Brazil. It is important to note that the instrument tested is quite broad and complex. It is therefore advisable to consider the possibility of future research and the inclusion of a larger number of participants when testing the model.
